# Correction: Relationship quality and mental health during COVID-19 lockdown

**DOI:** 10.1371/journal.pone.0257118

**Published:** 2021-09-01

**Authors:** Christoph Pieh, Teresa O´Rourke, Sanja Budimir, Thomas Probst

In the Abstract, there is an error in the eighth sentence of the first paragraph. The correct sentence is: Additionally, individuals without no relationship scored better on all scales except depression than individuals with poor relationship quality (all p-values < .05).

In the Results, there is an error in the second sentence of the fourth paragraph. The correct sentence is: In addition, individuals without relationship had better scores–in all scales except depression–than individuals with a poor relationship quality (all p < .05) (Table 4).

In the Discussion, there is an error in the fourth sentence of the third paragraph. The correct sentence is: Our result, that people with poor relationship quality showed more anxiety, even compared to people without relationships, is in contrast to the population-based study of Leach, Butterworth, Olesen, and Mackinnon [10], who reported that persons with poor relationship quality and singles had similar anxiety scores, results are however, comparable to Leach et al. for depression since singles and poor relationship did not differ from each other in our study as well.
