# Supplementary material for: Relationship quality and mental health during COVID-19 lockdown
Source: PLoS One. 2020 Sep 11;15(9):e0238906. doi: 10.1371/journal.pone.0238906 (PMC7485771; doi:10.1371/journal.pone.0238906)
Supplement: S1 Data — (DOCX) [file pone.0238906.s001.docx]

**Data presented in the manuscript (M ± SD)**

Comparisons between the three relationship groups regarding age and gender.

Relationship group

Good Poor No Total

relationship relationship relationship

quality quality

| **Age n (%)** | 18-24 | 53 (9.8) | 10 (5.3) | 55 (20.2) | 118 (11.7) |  |
| --- | --- | --- | --- | --- | --- | --- |
|  | 25-34  35-44 | 100 (18.4)  98 (18.0) | 32 (16.5)  47 (24.7) | 34 (12.5)  40 (14.7) | 166 (16.5)  185 (18.4) |  |
|  | 45-54 | 119 (21.9) | 41 (21.6) | 62 (22.8) | 222 (22.1) |  |
|  | 55-64 | 97 (17.9) | 38 (20.0) | 46 (16.9) | 181 (18.0) |  |
|  | 65+ | 76 (14.0) | 22 (11.6) | 35 (12.9) | 133 (13.2) |  |
| **Gender n (%)** | Male | 274 (50.5) | 93 (48.9) | 108 (39.7) | 475 (47.3) |  |

Female 269 (49.5) 97 (51.1)

Total 543 (100) 190 (100)

Results for depression, anxiety, insomnia, psychological quality of life, well-being, and perceived stress between relationship groups

Relationship group

Good Poor relationship No relationship Total

relationship

quality

| **PHQ-9 n (%)** | <10  >=10 | 470 (86.6)  73 (13.4) | 123 (64.7) 67 (35.3) | 201 (73.9) 71 (26.1) | 794 (79.0)  211 (21.0) |  |
| --- | --- | --- | --- | --- | --- | --- |
| **GAD-7 n (%)** | <10  >=10 | 476 (87.7)  67 (12.3) | 129 (67.9) 61 (32.1) | 209 (76.8%)  63 (23.2) | 814 (81.0)  191 (19.0) |  |
| **ISI n (%)** | <15 >=15 | 474 (87.3)  69 (12.7) | 148 (77.9) 42 (22.1) | 225 (82.7) 47 (17.3) | 847 (84.3)  158 (15.7) |  |

| **PHQ-9** | Total  M  SD | 543 (100)  4.87  4.78 | 190 (100)  8.41  5.40 | 272 (100)  7.25  5.83 | 1005 (100)  6.19  5.40 |  |
| --- | --- | --- | --- | --- | --- | --- |
| **GAD-7** | M SD | 4.91  4.29 | 7.86  4.77 | 6.28  4.92 | 5.84  4.70 |  |
| **ISI** | M SD | 7.46  5.42 | 10.17  5.98 | 8.69  5.74 | 8.31  5.70 |  |
| **WHOQOL BREF**  **psychological domain** | M SD | 75.43  16.01 | 60.16  18.34 | 65.40  20.02 | 69.83  18.70 |  |
| **WHO-5** | M SD | 16.42  4.81 | 12.35  5.25 | 14.20  5.76 | 15.05  5.40 |  |
| **PSS-10** | M SD | 14.28  6.91 | 19.12  7.13 | 17.15  7.85 | 15.97  7.47 |  |
